# Supplementary material for: NuMA recruits dynein activity to microtubule minus-ends at mitosis
Source: eLife. 2017 Nov 29;6:e29328. doi: 10.7554/eLife.29328 (PMC5706958; doi:10.7554/eLife.29328)
Supplement: Transparent reporting form [file elife-29328-transrepform.docx]

***eLife’s* transparent reporting form**

We encourage authors to provide detailed information *within their submission* to facilitate the interpretation and replication of experiments. If you have any questions, please contact us: [editorial@elifesciences.org](mailto:editorial@elifesciences.org).

**Sample-size estimation**

- You should state whether an appropriate sample size was computed when the study was being designed
- You should state the statistical method of sample size computation and any required assumptions
- If no explicit power analysis was used, you should describe how you decided what sample (replicate) size (number) to use

Please outline where this information can be found within the submission (e.g., page numbers or figure legends), or explain why this information doesn’t apply to your submission:

An appropriate sample size was not computed when the study was designed, and no explicit power analysis was used. For each experiment, sample size is indicated in the figure legend and was chosen and based on standards in the field and experimental limitations and variability.

**Replicates**

- You should report how often each experiment was performed
- You should include a definition of biological versus technical replication
- The data obtained should be provided and sufficient information should be provided to indicate the number of independent biological and/or technical replicates
- If you encountered any outliers, you should describe how these were handled
- Criteria for exclusion/inclusion of data should be clearly stated
- High-throughput sequence data should be uploaded before submission, with a private link for reviewers provided (these are available from both GEO and ArrayExpress)

Please outline where this information can be found within the submission (e.g., page numbers or figure legends), or explain why this information doesn’t apply to your submission:

The number of times that experiments were performed is included in the related figure legend. We discuss our definition of biological replication in the Statistics section of the Methods, and we provide sufficient information in our legends to understand the number of each type of replicate performed for a given experiment. When relevant, we explain our criteria for inclusion of data in the Methods (e.g., “To determine the percentage of p150 at plus-ends vs. minus-ends (Fig. 1B, Fig. 4C), we used single microtubules where both ends were clearly visible…”

**Statistical reporting**

- Statistical analysis methods should be described and justified
- Raw data should be presented in figures whenever informative to do so (typically when N per group is less than 10)
- For each experiment, you should identify the statistical tests used, exact values of N, definitions of center, methods of multiple test correction, and dispersion and precision measures (e.g., mean, median, SD, SEM, confidence intervals; and, for the major substantive results, a measure of effect size (e.g., Pearson's r, Cohen's d)
- Report exact p-values wherever possible alongside the summary statistics and 95% confidence intervals. These should be reported for all key questions and not only when the p-value is less than 0.05.

Statistical analysis methods are described in the Statistics section of the Materials and Methods. For each experiment as appropriate, we describe exact values of N and dispersion/precision measures (mean, SEM), effect size (Pearson’s r), and p-values in the Figure Legends.

Please outline where this information can be found within the submission (e.g., page numbers or figure legends), or explain why this information doesn’t apply to your submission:

(For large datasets, or papers with a very large number of statistical tests, you may upload a single table file with tests, Ns, etc., with reference to page numbers in the manuscript.)

**Additional data files (“source data”)**

- We encourage you to upload relevant additional data files, such as numerical data that are represented as a graph in a figure, or as a summary table
- Where provided, these should be in the most useful format, and they can be uploaded as “Source data” files linked to a main figure or table
- Include model definition files including the full list of parameters used
- Include code used for data analysis (e.g., R, MatLab)
- Avoid stating that data files are “available upon request”

Please indicate the figures or tables for which source data files have been provided:

We do not have additional source data files.
